# Antibiofilm activity from endophyte bacteria, *Vibrio cholerae* strains, and actinomycetes isolates in liquid and solid culture

**DOI:** 10.1186/s12866-023-02829-6

**Published:** 2023-03-29

**Authors:** Michael, Diana Elizabeth Waturangi

**Affiliations:** Faculty of Biotechnology, Atma Jaya Catholic University of Indonesia, Jalan Raya Cisauk-Lapan No. 10, Sampora, Cisauk, Tangerang, Banten 15345 Indonesia

**Keywords:** Antibiofilm, Culture conditions, Solid culture, Liquid culture, Endophyte vibrio cholerae, Actinomycetes

## Abstract

**Background:**

Biofilm-associated infections are a global threat to our economy and human health; as such, development of antibiofilm compounds is an urgent need. Our previous study identified eleven environmental isolates of endophyte bacteria, actinomycetes, and two strains of *Vibrio cholerae* as having strong antibiofilm activity, but only tested crude extracts from liquid culture. Here we grew the same bacteria in solid culture to induce the formation of colony biofilms and the expression of genes that may ultimately produce antibiofilm compounds. This research aimed to compare antibiofilm inhibition and destruction activities between liquid and solid cultures of these eleven environmental isolates against the biofilms of representative pathogenic bacteria.

**Results:**

We measured antibiofilm activity using the static antibiofilm assay and crystal violet staining. The majority of our isolates exhibited higher inhibitory antibiofilm activity in liquid media, including all endophyte bacteria, *V. cholerae* V15a, and actinomycetes strains (CW01, SW03, CW17). However, for *V. cholerae* strain B32 and two actinomycetes bacteria (TB12 and SW12), the solid crude extracts showed higher inhibitory activity. Regarding destructive antibiofilm activity, many endophyte isolates and *V. cholerae* strains showed no significant difference between culture methods; the exceptions were endophyte bacteria isolate JerF4 and *V. cholerae* B32. The liquid extract of isolate JerF4 showed higher destructive activity relative to the corresponding solid culture extract, while for *V. cholerae* strain B32 the solid extract showed higher activity against some biofilms of pathogenic bacteria.

**Conclusions:**

Culture conditions, namely solid or liquid culture, can influence the activity of culture extracts against biofilms of pathogenic bacteria. We compared the antibiofilm activity and presented the data that majority of isolates showed a higher antibiofilm activity in liquid culture. Interestingly, solid extracts from three isolates (B32, TB12, and SW12) have a better inhibition or/and destruction antibiofilm activity compared to their liquid culture. Further research is needed to characterize the activities of specific metabolites in solid and liquid culture extracts and to determine the mechanisms of their antibiofilm actions.

## Background

Biofilm-related infections are a major threat to the global economy and human health. Biofilms are assemblage of microorganism cells attached to biotic or abiotic surfaces and surrounded by extracellular matrix, and is the preferred mode of living for bacteria; 40–80% of prokaryotes live in such states [1]. However, from the human economic perspective, biofilms impose substantial costs; the economic burden of biofilms on medical and healthcare has been estimated at 386.8 billion US dollars annually [2]. Numerous diseases are associated with the formation of biofilms, including gastrointestinal diseases [3], gingivitis [4], otitis [5], cystic fibrosis [6], and chronic wounds [7]; moreover, biofilms contribute to 80% of chronic infections [8]. Functionally, biofilms provide microenvironments that change bacterial cell phenotypic features. The cells are enclosed in extracellular matrices that are mainly composed of exopolysaccharides, proteins, and extracellular DNA. These matrix function as a physical barrier against certain antibiotics, particularly for aminoglycoside antibiotics [9–11]; overall, cells inside the biofilm are 10–1000 × more resistant to killing by antimicrobial agents compared to their planktonic state [12, 13]. Additionally, persister cells within the biofilm contribute to the development of phenotypic tolerance against antimicrobial agents [14]. Failure to treat biofilm-related infections might lead to longer hospital stays, disability, and even death. Therefore, the development of antibiofilm compounds is an urgent need.

Microbial natural products have been a major source of scaffolds for drug therapies including compounds with anticancer, anti-inflammatory, immunosuppressive, antibiotic, and antibiofilm activities. Drug therapies from natural products usually target specific microorganisms and are less toxic to host cells as these compounds or metabolites are products of millions of years of evolution and natural selection [15]. Such specialized compounds and metabolites are important for microorganisms to compete and persist in the environment [16]. For example, an important aspect of competitive microbial interactions is the colonization of surfaces or places in a suitable environment [17]. In order to form a stable microbial colony, microorganisms use natural products or enzymes to clear and hold a space [17], such as rhamnolipid biosurfactant [18], cis-2-decenoic fatty acid [19], dispersin B [20], or group II capsular polysaccharides or exopolysaccharides [21]. These compounds represent promising leads for the development of antibiofilm drug therapies.

Here we used eleven bacteria that have demonstrated biofilm inhibition and/or elimination against a panel of pathogenic bacteria. Three types of isolates were tested, namely endophyte bacteria, *Vibrio cholerae*, and actinomycete*s*. The endophyte bacteria isolates were labeled AF1, JerF4, ShiF4, and BelF4; *Vibrio cholerae* isolates consisted of strain B32 and V15a; and the actinomycete*s* isolates comprised TB12, SW03, CW01, SW12, and CW17. We previously found these isolates to have strong antibiofilm activity; however, we originally only tested crude extracts from liquid culture. Research has shown that culture conditions can change the physiological state of bacteria and influence their production of natural products or metabolites. Here we also cultured the bacteria in solid culture to induce the formation of colony biofilms and also change the expression of genes that may induce production of antibiofilm compounds, particularly exopolysaccharides [22–25]. The aim of this research was to compare liquid and solid cultures of these eleven environmental isolates in terms of their antibiofilm inhibition and destruction activity against representative pathogenic bacteria.

## Results

### Solid and liquid culture conditions influence the biofilm inhibition activity of endophyte and *Vibrio cholerae* strains

To determine the inhibition activity of liquid or solid culture extracts, we used the 96-microtiter well plate method as described previously [26–28]. To assay inhibition activity, we added the extracts to the media at beginning of the incubation period. After incubation, the biofilms in the wells were stained using crystal violet and the absorbance was measured. We used a formula to calculate the inhibition activity (see Method), and applied Student’s *t*-test to determine any significant difference between liquid and solid culture extracts. To evaluate extract potency, we counted how many pathogen biofilms were significantly more inhibited by either the liquid extract or solid extract. If liquid extracts inhibited more pathogens than solid extracts, then the liquid extracts were considered more potent, and vice versa.

**Fig. 1:**
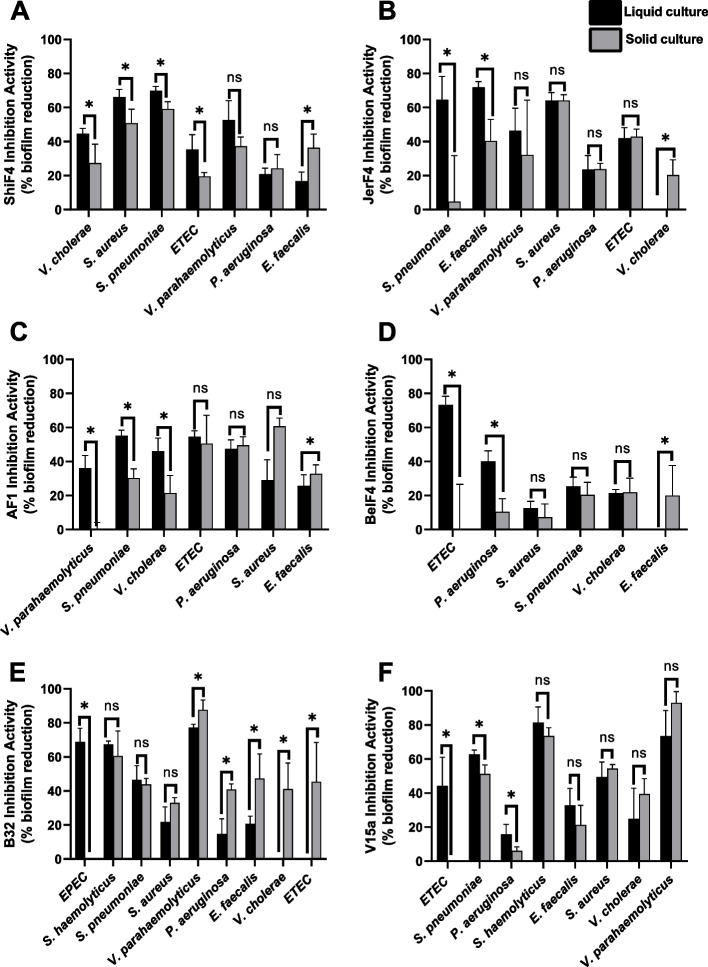
Biofilm inhibition activity from endophyte bacteria (**A-D**) and *Vibrio cholerae* strains (**E**–**F**) against the bacterial test panel. Extracts (liquid and solid extract) were added into the media with the concentration of 5% v/v. A statistical t-test was used to evaluate the mean values of antibiofilm activity between liquid and solid extract. Asterisks indicate activity values that are significantly different between liquid and solid crude extract (*, *P* < 0.05; ns, non-significant). If extracts didn’t inhibit biofilm formation the data were not shown in the graph

Overall, isolates from endophyte bacteria and *Vibrio cholerae* strains showed higher inhibition activity when they were incubated in liquid culture (Fig. 1). All endophyte bacteria strains (ShiF4, JerF4, AF1, and BelF4) showed higher inhibition activity for the liquid culture extract, inhibiting biofilm formation of two or more pathogens (Fig. 1 A**-**D). One *Vibrio cholerae* strain, V15a, also showed higher inhibition activity of its liquid culture extract, affecting the biofilms of three or more pathogenic bacteria (Fig. 1F); accordingly, the V15a liquid extract was considered more potent. On the other hand, isolate *Vibrio cholerae* B32 displayed higher inhibition activity of its solid culture extract against biofilm of five different pathogens (*V. parahaemolyticus*, *Pseudomonas aeruginosa, Enterococcus faecalis, V. cholerae,* and enterotoxigenic *Escherichia coli* (ETEC) (Fig. 1E) and hence the solid extract was considered more potent.

Interestingly, the antibiofilm inhibition activity of some extracts against certain pathogens was impaired according to the bacterial culture conditions. For example, the solid extracts from isolates JerF4 and BelF4 were respectively able to inhibit *V. cholerae* and *Enterococcus faecalis*, but corresponding liquid extracts did not have antibiofilm activity (Fig. 1B and D). Likewise, the solid extract of isolate B32 could inhibit *V. cholerae* and ETEC, whereas the liquid extract could not (Fig. 1E). Conversely, some antibiofilm activity was only observed in liquid extracts; for instance, those from endophyte bacteria AF1 and BelF4 inhibited biofilm formation by *V*. *parahaemolyticus* and ETEC, but not the solid extracts (Fig. 1C**-**D). Similarly, the liquid extracts of B32 and V15a inhibited the biofilms of ETEC and EPEC (enteropathogenic *Escherichia coli*) while corresponding solid extracts exhibited no activity (Fig. 1E**-**F).

### Extract source did not influence biofilm destruction activity for the majority of endophyte and *Vibrio cholerae* strains

As with inhibition activity, the biofilm destruction activity of compounds in liquid or solid culture extracts was determined using the 96-microtiter well plate method as described previously [26–28]. However, we added the extract to the media of the 24 h preformed biofilm and incubated it for 30 min to let the extract compounds interact with the biofilm. Afterwards, biofilms were stained using crystal violet and the absorbance measured. To calculate the inhibition activity, we used a formula (see Method), and significant differences between liquid and solid culture extracts were determined using Student’s *t*-test. To determine which extracts were more potent, we counted how many different pathogen biofilms were significantly more damaged by the liquid or solid extract; if more biofilms were damaged by the liquid culture extract, then that extract was considered more potent than the solid extract, and vice versa.

**Fig. 2:**
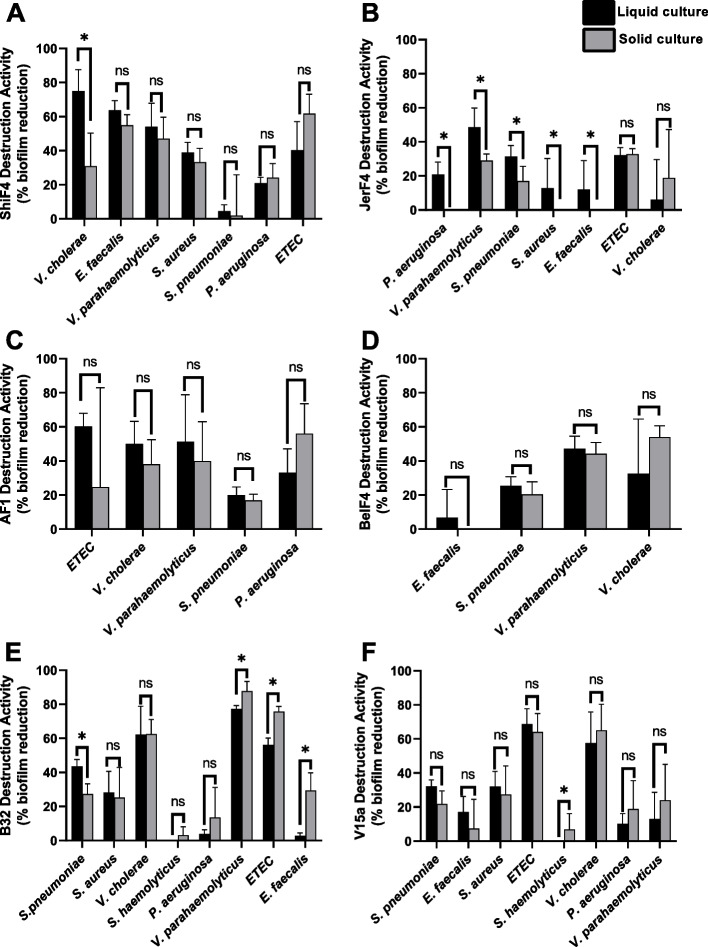
Biofilm destruction activity from endophyte bacteria (**A-D**) and *Vibrio ﻿cholerae* strains (**E**–**F**) against the bacterial test panel. Extracts (liquid and solid extract) were added to the media with the concentration of 5% v/v. A statistical t-test was used to evaluate the mean values of antibiofilm activity between liquid and solid extract. Asterisks indicate activity values that are significantly different between liquid and solid crude extract (*, *P* < 0.05; ns, non-significant). If extracts did not destroy biofilm the data were not shown in the graph

Overall, isolates from endophyte bacteria and *Vibrio cholerae* strains showed no significant difference in destruction activity between liquid and solid extracts (Fig. 2). Specifically, three isolates from endophyte bacteria (ShiF4, AF1, and BelF4) and the *Vibrio ﻿cholerae* isolate V15a showed no significant difference in destruction activity between solid and liquid culture (Fig. 2A, C, D, and F). Interestingly, the solid extract of isolate ShiF4 significantly inhibited *V. cholerae* biofilms, distinct from its liquid culture extract. Similarly, the solid extract of isolate V15a showed significant although weak destruction activity against *Staphylococcus haemolyticus* biofilm. Meanwhile, isolate JerF4 showed higher destruction activity when cultured in liquid culture against the biofilms of five different pathogenic bacteria (Fig. 2B), while the solid extract of isolate *Vibrio ﻿cholerae* B32 was able to destroy biofilms from three pathogenic bacteria (Fig. 2E).

### Solid and liquid culture conditions influence the antibiofilm inhibitory activity of actinomycetes strains

In our previous work [26–28], some selected actinomycetes strains were shown capable of inhibiting Gram-positive and Gram-negative bacteria and potentially producing potent antibiofilm compounds. In this study, we picked five actinomycetes strains and tested them against two bacterial pathogens: *Streptococcus pneumoniae* as a representative Gram-positive bacterium, and *Pseudomonas aeruginosa* as a representative Gram-negative bacterium. To determine the inhibition activity of compounds in liquid or solid culture extracts, we used the 96-microtiter well plate method as described in previous studies [26–28]. Actinomycetes attach to the media agar while growing in solid culture, so that we extracted compounds from the agar medium. These extracts were added to wells at the beginning of the incubation period. After incubation, the biofilms were stained using crystal violet and the absorbance was measured. To calculate inhibition activity, we used a formula (see Method); we then applied Student’s *t*-test to identify significant differences between liquid and solid culture extracts. To determine which extracts were more potent, we counted how many different pathogen biofilms were significantly more inhibited by either the liquid or solid extract. If the liquid extract inhibited more pathogens than the solid extract, the liquid extract was considered more potent, and vice versa.

**Fig. 3:**
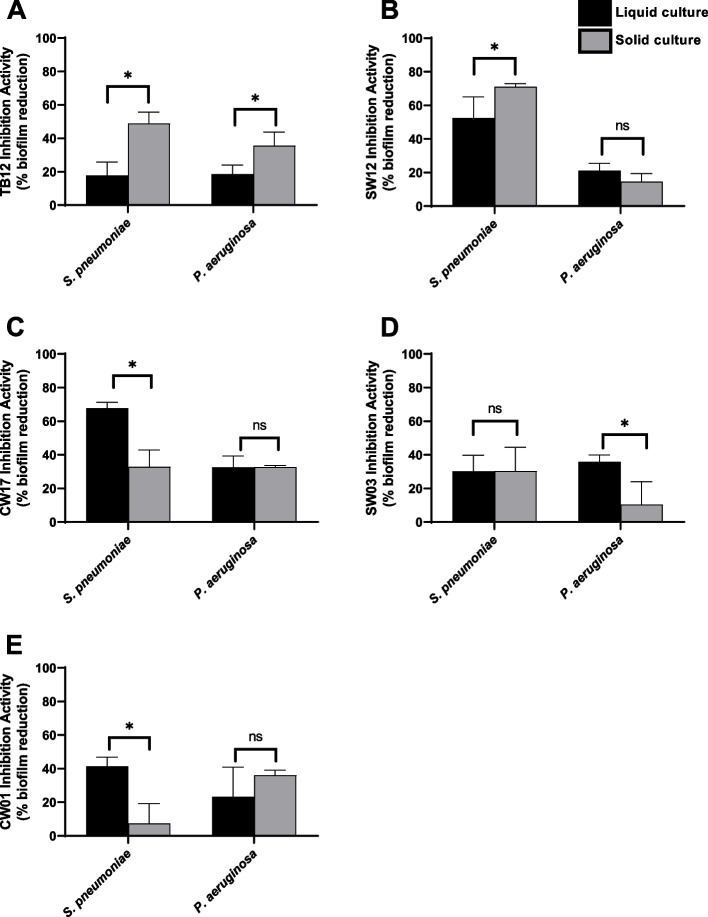
Biofilm inhibition activity from actinomycetes (**A-E**) against *S. pneumoniae* and *P. aeruginosa*. Extracts (liquid and solid extract) were added into the media with the concentration of 5% v/v. A statistical t-test was used to evaluate the mean values of antibiofilm activity between liquid and solid extract. Asterisks indicate activity values that are significantly different between liquid and solid crude extract (*, *P* < 0.05; ns, non-significant)

Culture conditions were found to influence the inhibition activity of some actinomycetes strains. In particular, solid extract from TB12 showed enhanced activity against both pathogenic bacteria, while that from SW12 showed greater activity against *S. pneumoniae* only (Fig. 3 A-B). Liquid extracts from the two strains, CW17 and CW01, showed higher inhibition activity only against *S. pneumoniae*; activity against the Gram-negative *P. aeruginosa* was not affected by the type of media (Fig. 3 C and E). Meanwhile, the liquid extract from isolate SW03 showed higher antibiofilm inhibition activity against the Gram-negative bacterium *P. aeruginosa* but not against *S. pneumoniae* (Fig. 3 D).

## Discussion

The production of metabolites in bacteria is greatly dependent on growth conditions or media type; as such, it is important to find the optimal conditions for producing specific compounds. In previous research, some bacteria that we tested were found able to inhibit or destroy biofilms, in particular endophyte bacteria, *Vibrio ﻿cholerae* strains, and actinomycetes [26–28]. These three groups are prolific producers of secondary metabolites, including compounds with antibiofilm activity [29–34]. However, we only used extracts from one growth condition, namely liquid culture or broth. Culturing bacteria in solid media or agar plates has been reported to change their metabolism, lifestyle, and gene expression, which changes lead to the production of compounds not detected in the supernatant of the liquid culture [35–38]. Here we cultured endophyte bacteria, *Vibrio ﻿cholerae* strains, and actinomycetes in liquid and solid media, extracted compounds from both conditions, and compared the antibiofilm activities of the extracts against a panel of pathogenic bacteria.

The results indicated that solid media can enhance the antibiofilm activity of some isolates. In particular, three isolates showed higher biofilm inhibition or destruction activity when cultured on solid media; these included *Vibrio ﻿cholerae* strain B32 and two actinomycetes bacteria (TB12 and SW12). Generally, solid media provide a surface to which bacteria can attach and thereby facilitates the development of high cell numbers in a limited space [39]. Such conditions increase interaction between cells and might in turn induce the production of metabolites; in particular, this culture mode can provide growth conditions that are similar to a biofilm. The colony biofilm microenvironment can increase interaction and communication between cells via quorum sensing, which might lead to the activation of silent biosynthetic gene clusters [39, 40].

Notably, solid media might affect the production of secondary metabolites of actinomycetes. Actinomycetes is a prolific producer of secondary metabolites that produced many compounds including antibiotics, anticancer, and antiparasitic [41]. They have a complex life cycle and produce secondary metabolites during a specific developmental stage [42]; moreover, the actinomycete *Streptomyces ceolicolor* has been shown to have different transcriptome profiles in liquid and solid cultures, with genes related to the production of a hydrophobic coat and spore maturation being upregulated in solid culture [43]. Similar to fungi, actinomycetes grow on or in the soil and adapted to live as terrestrial [44]. Therefore the cultivation of actinomycetes strains in liquid culture might not be inducing some of the biosynthetic gene clusters that are triggered in their natural environment [44]. The results of the present study suggest that solid media might induce the production of metabolites that enhance antibiofilm activity in two actinomycetes isolates (TB12 and SW12). Those strains might be suitable for solid state fermentation. Solid state fermentation (SSF) has been used to produce metabolites from actinomycetes and fungi species because they provide solid support and in some cases induce specific metabolites [45, 46]. SSF can increase the production yield of hygromycin by *Streptomyces spadicus* more than 4000 fold and on top of that also enhance the production of nogalamycin, the iron-binding siderophore, and rimocidin by various actinomycetes species [46]. Similar to actinomycetes, some metabolites only produces in SSF. A metabolomic study using *Penicillium expansum* KACC 40815 also found that some fungal metabolites, including meroterpenoid compounds, are only produced when growing in the solid state fermentation condition [47]. Although the study is not conducted using bacteria but that provide an example that solid media can induces the production of secondary metabolites. Ultimately, small change in growth condition like growing the bacteria on solid medium can enhance the production of antibiofilm compounds by some actinomycetes solid culture.

This study also found that culture type can influence the antibiofilm activity of *Vibrio* strains. *Vibrio cholerae* strains are able to form a biofilm [48] and solid media can provide a surface for the bacteria to attach to where they form microcolonies and promote the formation of colony biofilms [49]. Colony biofilm refers to the biomass of bacterial cells grown on agar media or on a semipermeable membrane on a solid culture [50]. Colony biofilms have been shown to contain compounds that can regulate biofilm development and dispersion [35–37]; moreover, bacterial cells in a colony biofilm exhibit different transcriptome [51–54], metabolome [38], and proteome [12] profiles due to being exposed to nutrient deprivation, oxygen limitation, and a higher level of soluble factors. Here we found one *Vibrio cholerae* strain (B32) that exhibited higher antibiofilm activity when grown in solid culture. This strain might produce compounds inside the colony biofilm that are not normally produced when living in the planktonic stage. It has been reported that polysaccharides are elevated in biofilms [22–25], and one study suggested that *Vibrio* sp. can produce an exopolysaccharide capable of inhibiting or eradicating biofilms. We infer that *Vibrio* strain B32 might be producing polysaccharide compounds; however, further investigation and characterization remains needed. Nonetheless, compounds from the solid culture of this *Vibrio* strain are potentially valuable for the development of new antibiofilm agents and merit further study.

Despite the above findings, we did not observe solid media to enhance antibiofilm activity for the majority of the tested isolates; rather, antibiofilm activity was more often higher in liquid cultures. This applied in four isolates from endophyte bacteria (AF1, BelF4, JerF4, and ShiF4), the *Vibrio* strain V15a, and three isolates from actinomycetes (CW17, SW03, and CW01). One reasons for this finding is that we selected isolates which exhibited decent antibiofilm activity in liquid culture in previous research [26]. Several studies have reported that isolates having decent antibiofilm activity when cultured in solid media exhibited poor antibiofilm activity in corresponding supernatant or liquid media [35–37]. Polysaccharides that accumulate or are produced within colony biofilms are just one of the types of compounds responsible for antibiofilm activity [35–37]. As we did not characterize the compounds in these crude extracts, it is possible they were not polysaccharides and did not accumulate in the biofilm. Rather, the bacteria might release soluble compounds into the media. Nonetheless, as endophyte bacteria are well known to produce many compounds to inhibit biofilm formation, including quorum quenching agents, our results align with the literature. For example, endophyte bacteria produce AHL acylase [55], AHL lactonase [56], and DSF-degrading enzyme [57] that they excrete into the environment or media.

One isolate of *Vibrio* V15a from our study showed higher antibiofilm activity in liquid culture. It is likely that this isolate also secretes compounds, which aligns with previous studies that used supernatants from *Vibrio* sp. QY101 as a source of exopolysaccharide A101 to inhibit biofilm formation by *P. aeruginosa* [30] and from *Vibrio* MO245 as a source of a hyaluronan-like exopolysaccharide [58]. Another study also reported the strain *V. natriegens* MK3 to produce a biosurfactant in the supernatant that inhibits formation of biofilms by *V. harveyi* [59]. Production of such compounds might explain why *Vibrio* V15a has higher activity in the supernatant in comparison to solid culture.

Some actinomycetes isolates in our study also showed high activity when grown in liquid culture. It has previously been reported that actinomycetes, namely *Streptomyces ceolicolor*, have different transcriptomic profiles when cultured in solid and liquid media, with nearly 14% of genes being differentially expressed [43]. Genes upregulated in liquid culture included those for the production of specialized metabolites relating to calcimycin, tylosin A, tylosin D, calcium-dependent antibiotics, yellow antibacterial pigment, coelichelin, and desferrioxamine [43, 46]. The results from our study are consistent with this and several other studies that have shown liquid media to trigger the production of secondary metabolites in some actinomycetes strains [60–62].

In general there are differences in antibiofilm activity between solid and liquid culture. As a small change of cultivation parameters can alter the profile of the metabolome of bacterial species [63]. However, it is strains dependent. Microbes adapt and respond to the cues in the environment to thrive in natural habitat by modifying their transcriptome, and proteome which impact their metabolome [64]. Some strains will change metabolism and others will not.In some strains in this study, the production of compounds might not be not affected by solid or liquid culture; rather, the metabolites might be constitutively produced regardless of growth condition. One study showed some genes related to the production of secondary metabolite to have comparable transcript abundances in different growth conditions, for example, the gene for production of erythromycin A, actinorhodin and undecylprodigiosin [43, 46]. The results from our study are likewise consistent with prior results indicating that medium type does not affect the production of some secondary metabolites in some strains.

## Limitation

We only tested a select set of isolates in this research; as such, our data do not represent all the bacterial species that can be grown in solid or liquid media and cannot be applied to decide which medium is best for the production of antibiofilm compounds. More research is required with larger sample sizes, as well as the microscopic determination of antibiofilm activities. In addition, we only used crude extracts in this study. One strain can produce numerous secondary metabolites or compounds, as it might harbor silent biosynthetic gene clusters [65]. Further research is needed to purify the antibiofilm compounds and quantify their concentrations in each extract. Lastly, the results from the destruction evaluation are mostly non-significant, which might be because we used a crystal violet assay. In future testing of compounds, we can try other methods like Calgary devices so as to improve the results [66].

## Conclusion

In this study, we showed solid and liquid culture conditions to influence the antibiofilm activity of crude extracts from some isolates against our bacterial test panels. We used the static antibiofilm assay and crystal violet staining to assess antibiofilm inhibition and destruction activities of the extracts. Although the majority of isolates exhibited better antibiofilm activity in liquid culture extracts, we found solid extracts from *Vibrio ﻿cholerae* strain B32 and the two actinomycetes TB12 and SW12 to show higher antibiofilm activity. These findings support that for some bacteria, culture condition influences the production of secondary or specialized metabolites that have antibiofilm effects against the pathogenic bacteria in the test panel. Thus, culture condition is one parameter that can be used to optimize the production of compounds by bacterial species or in larger-scale production. Further study is needed to characterize the metabolites in these extracts, determine the mechanism of action of the active fraction, and observe the effect of bioactive compounds on bacterial biofilms using confocal microscopy experiments.

## Materials and method

### Bacterial isolates and culture conditions

Bacterial isolates from previous study were used in this research were listed in Table 1, including five isolates of soil sediment actinomycetes [26], four isolates of endophyte bacteria, and two strains of *Vibrio cholerae*. Actinomycetes isolates were cultured on GYMS Agar (malt extract 10 g, yeast extract 4 g, agar 12 g, CaCO3 2 g, glucose 4 g, starch 20 g and ddH2O 1000 ml) and incubated at 28 °C for 7 days. Endophyte isolates were cultured on Brain Heart Infusion Agar (Oxoid) and incubated at 37 °C overnight. *Vibrio cholerae* isolates were refreshed on Thiosulfate-citrate-bile salts-sucrose agar (Oxoid) and incubated at 37 °C overnight. Eight pathogenic bacteria were used in this research are listed in Table 2. Pathogenic bacteria of *V. parahaemolyticus* and *V. cholerae* were maintained on thiosulfate citrate bile salt (Oxoid) agar at 37 °C for overnight. All other isolates were maintained on brain heart infusion agar (Oxoid) at 37 °C overnight.

**Table 1:** A list of antibiofilm-producing isolates and their origin

Bacterial Strains	Origin
*Arthrobacter mysorens* TB12	Lake sediment, Telaga Biru, Cibodas
*Streptomyces sp.* SW03	Paddy field sediment, Sawah, Gancahan 8 village, Sleman
*Arthrobacter sp.* CW01	River sediment, Cunca Wulang, West Flores
*Streptomyces sp.* CW17	River sediment, Cunca Wulang, West Flores
*Streptomyces carpaticus* SW12	Paddy field sediment, Gancahan 8 village, Sleman
*Bacillus amyloliquefaciens* AF1	*Anredera cordifolia*, Jakarta
*Pseudomonas putida* BelF4	*Pluchea indica*
*Pseudomonas psychrotolerans* ShiF4	*Piper betle*
*Lysinibacillus fuciformis* JerF4	*Citrus* sp.
*Vibrio cholerae* B32	Ice cube, Jakarta
*Vibrio cholerae* V15a	Ice cube, Jakarta

**Table 2:** A list of bacterial test panel

Pathogenic bacteria	Origin
*Staphyloccocus aureus*	ATCC 29213
*Streptococcus pneumoniae*	ATCC 49616
*Enterococcus faecalis*	ATCC 33186
*Staphyloccocus haemolyticus*	ATCC 29970
*ETEC* usn* (Enterotoxigenic Escherichia coli)*	US Namru
*Pseudomonas aeruginosa*	ATCC 27853
*Vibrio cholerae*	Ice cube, Jakarta
*Vibrio parahaemolyticus*	ATCC 17802
*EPEC* usn* (Enteropethogenic Escherichia coli)*	US Namru

### Production of crude extracts in liquid media

The production was conducted using a liquid medium in Erlenmeyer flasks for endophyte bacteria and *Vibrio cholerae* strains in. One loop of bacteria was inoculated into 20 mL of brain heart infusion broth (Oxoid) as a production medium and incubated at 37 °C with 120 rpm agitation for 24 h. While for actinomycetes, the isolate was inoculated into 20 mL tryptone soya broth (Oxoid) as a production medium and incubated at 28 °C with agitation at 120 rpm for 7 days as instructed in the previous study [26]. The culture was transferred into centrifuge tubes and centrifuged at 7798 RCF (Thermo Scientific) for 10 min. The supernatant was collected and transferred to centrifuge tubes, and subsequently centrifuged again at 7798 RCF (Thermo Scientific) for 10 min. After the second centrifugation, the supernatant was collected and used for the antibiofilm assays or stored at 4 °C for a week or at -20 °C for a month.

### Production of crude extracts in solid media

The production process of solid crude extracts from endophyte bacteria and *Vibrio cholerae* isolates was done on agar plates. One loop of bacteria was suspended in 100 *µ*L of liquid medium and spread by glass spreader into 20 mL of brain heart infusion broth (Oxoid) + 2% of agar (Oxoid) as production medium, and incubated at 37 °C for 24 h. Colony biofilm was scraped from the agar plate and transferred to 20 mL of physiological salt solution or 0.85% of NaCl [36, 37]. While for actinomycetes isolate, 100 µL of liquid suspension was spread by glass spreader into 20 mL of tryptone soya broth (Oxoid) + 2% of agar (Oxoid) as production medium and incubated at 28 °C for 7 days. Culture on the agar plate was chopped using a scalpel into small pieces and transferred to 20 mL of physiological salt solution or 0.85% of NaCl. All tubes containing solid culture were homogenized with vortex for 10 min. The tube was centrifuged at 7798 RCF (Thermo Scientific) for 10 min and transferred to centrifuge tubes, and subsequently centrifuged again at 7798 RCF (Thermo Scientific) for 10 min. After the second centrifugation, the supernatant was collected and used for the antibiofilm assays or stored at 4 °C for a week or at -20 °C for a month.

### Static inhibition and destruction biofilm assay in 96-microtiter well plate

The pathogenic bacteria were inoculated into glass test tubes containing 1% of glucose-brain heart infusion medium (Oxoid) and incubated at 37 °C overnight. The cultures were adjusted to OD600 of 0.132 or a McFarland value of 0.5. Next, 200 *µ*L of pathogenic bacteria suspension were inoculated into each well of the microplate (Iwaki). For the Inhibition assay, 20 *µ*L of solid or liquid crude extracts were added to the well and incubated for 24 h. While for destruction assay, biofilms were developed in a microplate (Iwaki) by incubating the plate for 24 h, then the mature biofilms were co-incubated with 20 *µ*L of solid or liquid crude extracts for 30 min at 37 °C. After the incubation, planktonic cells and spent media were discarded and adherent cells were rinsed twice using sterile distilled water and air dried. Adherent biofilm was then stained with 0.4% of crystal violet solution for 30 min and subsequently rinsed five times using distilled water and air dried. Crystal violet was then solubilized with 200 *µ*L of ethanol absolute (Merck). The solubilized crystal violet was transferred to a new microplate, and the optical density was determined at 595 nm using a microplate reader (Biorad) [67]. All experiments were performed at least four times. The percentage of biofilm reduction activity were calculated using formula:

$$\%\;\mathrm B\mathrm i\mathrm o\mathrm f\mathrm i\mathrm l\mathrm m\;\mathrm r\mathrm e\mathrm d\mathrm u\mathrm c\mathrm t\mathrm i\mathrm o\mathrm n=1-\frac{\mathrm{OD}\;\mathrm{sample}-\mathrm{OD}\;\mathrm{negative}\;\mathrm{control}}{\mathrm{OD}\;\mathrm{positive}\;\mathrm{control}-\mathrm{OD}\;\mathrm{negative}\;\mathrm{control}}\ast100\%$$

### Statistical analysis

The results were shown as mean ± SD (Standard Deviations) and were analysed by a student’s t-test. A *P*-value of ≤ 0.05 was statistically significant. The figures were created using GraphPad Prism software, V. 7.0.

## Data Availability

The 16S rRNA gene sequences of the isolates have been submitted to the NCBI genebank database (Home—PopSet—NCBI (nih.gov). *Streptomyces sp.* SW03 JX434841 (Actinomycetia 16S ribosomal RNA gene, partial sequence.—PopSet—NCBI (nih.gov), *Arthrobacter mysorens* TB12 JX434842 (Actinomycetia 16S ribosomal RNA gene, partial sequence.—PopSet—NCBI (nih.gov), *Arthrobacter sp.* CW01 JX434848, *Streptomyces sp.* CW17 JX434845 Actinomycetia 16S ribosomal RNA gene, partial sequence.—PopSet—NCBI (nih.gov), *Streptomyces carpaticus* SW12 JX434849, *Bacillus amyloquefaciens* AF1OP458755, *Pseudomonas psychotolerans* ShiF4 OP458791, *Lysinibacillus fuciformis* JerF4 OP458783.

## Abbreviations

AHL: Acyl-homomserine lactones
DNA: Deoxyribonucleic acid
DSF: Diffusible signal factor
ETEC: Enterotoxigenic *E. coli*
EPEC: Enteropathogenic *E. coli*
GYMS: GYM Streptomyces Medium
RCF: Relative Centrifugal Force
